# Activation of Protein Kinases and Phosphatases Coupled to Glutamate Receptors Regulates the Phosphorylation State of DARPP32 at Threonine 75 After Repeated Exposure to Cocaine in the Rat Dorsal Striatum in a Ca^2+^-Dependent Manner

**DOI:** 10.1093/ijnp/pyv075

**Published:** 2015-07-04

**Authors:** Jieun Kim, In Soo Ryu, Su Yeon Seo, Eun Sang Choe

**Affiliations:** Department of Biological Sciences, Pusan National University, Pusan, Korea.

**Keywords:** DARPP32, glutamate, phosphorylation and dephosphorylation, psychostimulant, striatum

## Abstract

**Background:**

Phosphorylation state of dopamine- and cAMP-regulated phosphoprotein, molecular weight 32kDa (DARPP32) is crucial to understand drug-mediated synaptic plasticity. In this study, mechanisms underlying repeated cocaine-stimulated phosphorylation of DARPP32 at threonine 75 (pDARPP32-Thr75) were determined by investigating the hypothesis that activation of protein kinases and phosphatases coupled to glutamate signaling is necessary for the regulation of pDARPP32-Thr75 after repeated cocaine administration.

**Methods:**

Intracaudate drug infusions into the rat dorsal striatum followed by Western immunoblot analysis were mainly performed to test this hypothesis.

**Results:**

The results demonstrated that 7 repeated daily intraperitoneal injections of cocaine (20mg/kg) upregulated the expression of pDARPP32-Thr75. Increases in the cytosolic Ca^2+^ concentrations followed by Ca^2+^-dependent protein kinase activation through stimulation of Ca^2+^ channels in striatal neurons were necessary for the phosphorylation. Activation of protein phosphatases further regulated the phosphorylation state by deactivating pDARPP32-Thr75 and upstream protein kinases.

**Conclusion:**

These findings suggest that activation of protein kinases and phosphatases coupled to glutamate receptors controls the phosphorylation state of DARPP32-Thr75 after repeated exposure to cocaine in the dorsal striatum in a Ca^2+^-dependent manner.

## Introduction

Cocaine administration increases extracellular dopamine concentrations by blocking dopamine reuptake at its terminal in the dorsal striatum and acts as an indirect dopamine agonist (Post and Rose, 1976; Ritz et al., 1987; Koob and Bloom, 1988). This increase then potentiates the release of glutamate through transsynaptic activation of basal ganglia circuitry (Kalivas, 2004). Stimulation of both ionotropic- and metabotropic glutamate receptors (mGluRs) leads to extracellular Ca^2+^ influx into the gamma amino-butyric acid (GABA) neurons of the dorsal striatum (Nakanishi et al., 1998). Repeated exposure to cocaine also facilitates Na^+^ influx through stimulation of α-amino-3-hydroxy-5-methyl-4-isoxazolepropionic acid (AMPA) and kainate glutamate receptors, through which Ca^2+^ release from the internal Ca^2+^ store is further regulated (Choe et al., 2006; Kim et al., 2009). Increases in Ca^2+^ mobilization into the cytosol then trigger activation of Ca^2+^-dependent signaling cascades, leading to short- and long-term changes in physiology and behavior in response to cocaine exposure (Gnegy, 2000; Easton et al., 2014).

Dopamine- and cAMP-regulated phosphoprotein, molecular weight 32kDa (DARPP32) is highly expressed in GABAergic neurons of the dorsal striatum (Ouimet et al., 1984). DARPP32 has been found to contain multiple phosphorylation sites that are determined by protein kinases linked to dopamine and glutamate receptors (Svenningsson et al., 2004). Activation of protein kinase A (PKA) coupled to dopamine D1 receptors phosphorylates the threonine (Thr) residue at position 34 of DARPP32, which potentiates dopamine D1 receptor-mediated signaling cascades (Hemmings et al., 1984). While it inhibits protein phosphatase 1 (PP1) and causes the sustained phosphorylation of GluR1 subunit of AMPA receptors, resulting in the potentiation of glutamatergic transmission in the dorsal striatum (Matsuyama et al., 2003). Additionally, activation of cyclin-dependent kinase 5 (CDK5) after glutamate receptor stimulation phosphorylates DARPP32 at Thr75 (pDARPP32-Thr75), which acts as a PKA inhibitor in mice (Bibb et al., 1999; Greengard, 2001). Evidence has shown that stimulation of the group 1 mGluRs with DHPG increased the expression of pDARPP32-Thr75 in the neostriatal slice; however, the blockade of them with L-AP3 did not (Liu et al., 2001). Taken together, these findings suggest that the phosphorylation state of DARPP32 is regulated by the interactions of dopamine- and glutamate receptor-coupled signaling cascades. Based on these findings, the synaptic plasticity associated with DARPP32 seems to be controlled by protein kinase-specific phosphorylation in response to a variety of stimuli, including drug exposure.

Previous studies have shown that acute and repeated cocaine administration phosphorylates Thr34 and Thr75 of DARPP32 in mice through PKA and CDK5 activation, respectively (Nishi et al., 2000; Bibb et al., 2001). Cocaine sensitization increases pDARPP32-Thr75 in the caudate-putamen and the nucleus accumbens of rats (Scheggi et al., 2004). Taken together, these findings suggest that DARPP32 is a key regulator of drug addiction that acts in a phosphorylation site-dependent manner, which is closely associated with dopamine and glutamate receptors. Thus, in this study, we investigated whether activation of protein kinases and phosphatases linked to glutamate receptors is necessary for the regulation of pDARPP32-Thr75 in the dorsal striatum, a structure closely associated with drug addiction by integrating dopaminergic and glutamatergic transmission, after repeated cocaine administration.

## Materials and Methods

### Animals

Adult male Sprague-Dawley rats (210–250g) were obtained from Hyo-Chang Science Co. (Daegu, Korea). Rats were separated into pairs and habituated for 3 days in home cages. After acclimation, the rats were handled for 3 days. Food and water were provided ad libitum, and the rats were maintained on a 12 h light/ dark cycle throughout the experimental period. In addition, temperature and humidity were maintained at 21°C to 23°C and 45 to 55%, respectively. Experiments were performed in a quiet room to minimize stress to the animals. All animal use procedures were approved by the Institutional Animal Care and Use Committee and were conducted in accordance with the provisions of the NIH Guide for the Care and Use of Laboratory Animals.

### Drugs

All pharmacological drugs, except cocaine (Balgopia, Louvain-La-Neuve, Belgium), were purchased from Tocris Bioscience (Bristol, UK) and working solutions were always prepared fresh for experiments. The Ca^2+^ chelator, BAPTA-AM (50 nmol), the noncompetitive *N*-methyl-D-aspartate (NMDA) receptor antagonist, MK801 (2 nmol), the competitive NMDA receptor antagonist, DL-AP5 (2 nmol), the L-type voltage operated Ca^2+^ channel blocker, nifidipine (60 nmol), the selective P- and N-type calcium channel blocker, ω-Agatoxin IVA (Agatoxin IVA) (30 pmol for P-type; 100 pmol for N-type), the Na^+^ channel blocker, tetradotoxin citrate (TTX) (1 pmol), the ryanodine Ca^2+^ channel blocker, dantrolene (20 nmol), the trisphosphate inositol (IP_3_)-sensitive Ca^2+^ channel blocker, xestosponin C (0.004 nmol), the potent Ca^2+^ and calmodulin protein kinase II (CaMKII) inhibitor, KN93 (10 nmol), the selective mitogen-activated protein kinase inhibitor for extracellular-regulated kinase 1/2 (ERK1/2), SL327 (150 nmol), the potent and selective protein kinase C (PKC) inhibitor, GF109203X (20 nmol), the selective c-Jun N-terminal kinase (JNK) inhibitor, SP600125 (200 nmol), the potent and selective PKA inhibitor, KT5720 (5 nmol), the selective PP1/2A inhibitor, okadaic acid (0.5 nmol) or the potent PP2B inhibitor, cyclosporin A (0.5 nmol), was dissolved in the minimum concentration of dimethylsulfoxide (DMSO) and then diluted in artificial cerebro-spinal fluid (aCSF) containing (mM) 123 NaCl, 0.86 CaCl_2_, 3.0 KCl, 0.89 MgCl_2_, 0.50 NaH_2_PO_4_, and 0.25 Na_2_HPO_4_ aerated with 95% O_2_/5% CO_2_, pH 7.2 to 7.4 or NaCl. The same DMSO-aCSF mixture solution was used as the vehicle control for a given drug. The drug solutions were all adjusted to neutral with NaOH if necessary. Rats were received repeated intraperitoneal (i.p.) injections of saline or cocaine (20mg/kg) once per day for 7 consecutive days. Cocaine was injected in a volume of 1mL in physiological saline (0.9% sodium chloride). The concentrations of drugs were determined from previous studies (Ahn and Choe, 2009, 2010; Oh et al., 2013; Yang and Choe, 2014) except for KN93 (Anderson et al., 2008).

### Surgery and Intracaudate Drug Infusion

**Figure 1.**
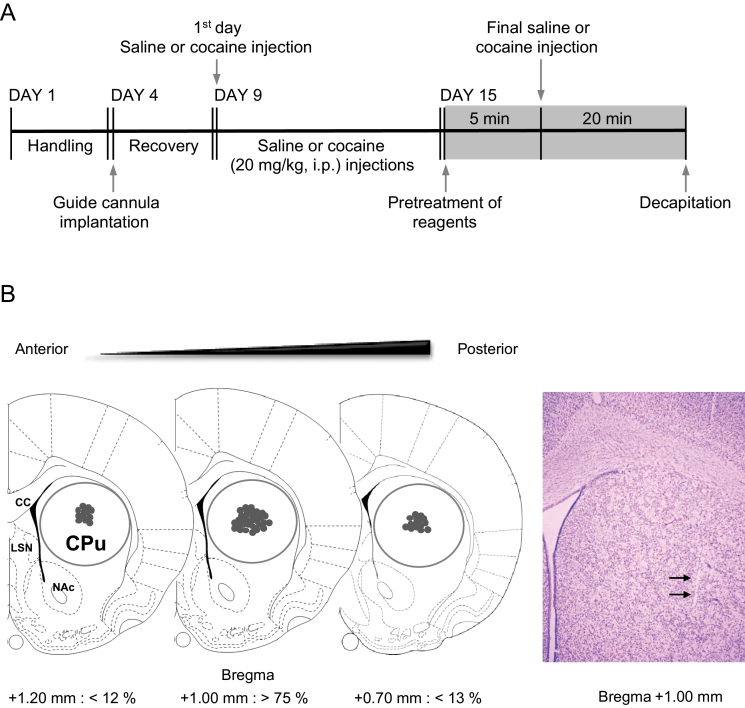
Schematic diagram illustrating the experimental schedule. All reagents were microinjected into the right dorsal striatum (CPu) 5 minutes prior to the final injection of saline or cocaine. (A) Gray shadow represents the final day of each experiment. Brain sections showing the reconstruction of microinjection placements and area for the analysis of Western immunoblotting (circles) in the CPu. (B) A Nissl-stained photomicrograph including a microinjection (arrows) shows that gliosis was not occurred by the implantation of guide cannula and the infusion of drugs. cc, corpus callosum; LSN, lateral septal nuclei; NAc, nucleus accumbens.

Rats were anesthetized with 8% chloral hydrate (5.8mL/kg, i.p.) then placed in a stereotaxic apparatus. Under aseptic conditions, a 23-gauge stainless steel guide cannula (0.29 mm inner diameter, 10mm in length) was implanted 1mm anterior to the bregma, 2.5mm right of the midline, and 5mm below the surface of the skull. The guide cannula was sealed with a stainless-steel wire of the same length. The rats were then allowed to recover from surgery for 5 days prior to the experiment. On the day of the experiment, the inner steel wire was replaced with a 30-gauge stainless steel injection cannula (0.15 mm inner diameter, 12.5mm in length) that protruded 2.5mm beyond the guide cannula. Throughout the experiments, all drugs were infused unilaterally into the central part of the right dorsal striatum 5 minutes prior to the final i.p. injection of cocaine or saline in a volume of 1 μL at a rate of 0.2 μL/min in freely moving rats (Figure 1A). The progress of the injection was monitored by observing the movement of a small air bubble along the length of precalibrated PE-10 tubing inserted between the injection cannula and a 2.5 μL Hamilton microsyringe (Fisher Scientific, Pittsburgh, PA). After completing the injection, the injector was left in place for an additional 5 minutes to reduce any possible backflow of the solution along the injection tract. The physical accuracy of the injection was verified by the reconstruction of microinjection placements (Figure 1B). The possibility of gliosis caused by the implantation of the guide cannula and the infusion of drugs dissolved in DMSO/aCSF was verified by Nissl staining (Figure 1B).

### Western Immunoblotting

Western immunoblotting was routinely performed and repeated it more than 3 times as previously described (Choe et al., 2004). Briefly, the rats were deeply anesthetized with 8% chloral hydrate, then decapitated 20 minutes after the final drug injection. Next, the brains were removed, frozen in isopentane at -70°C, and stored in a deep freezer until used. Sections were serially cut using a cryostat (Leica biosystems, Nussloch, Germany) at -25°C, after which the injected right dorsal striatum was removed with a steel borer (2 mm inner diameter) (Figure 1B). All tissue samples were transferred to homogenization buffer containing (mM) 10 Tris-HCl, pH 7.4, 5 NaF, 1 Na_3_VO_4_, 1 EDTA, and 1 EGTA, sonicated 3 times for 9 seconds each, and then incubated on ice for 1 hour. After sonicating, samples were centrifuged at 13000rpm for 30 minutes at 4°C. The pellet, which primarily contains nuclei and large debris, was discarded, and the supernatant was centrifuged again at 13000rpm for 30 minutes at 4°C. The concentration of the solubilized proteins in the supernatant fraction was determined based on the Bradford method using a Bio-Rad Protein Assay (Bio-Rad Laboratories, Hercules, CA). The proteins in the supernatant were resolved using 10% sodiumdodecyl sulfate-polyacrylamide gel electrophoresis, after which the separated proteins were transferred to a nitrocellulose membrane. Next, the membrane was blocked with blocking buffer containing 5% skim milk in a mixture of Tris-buffered saline and Tween-20 (TBST), then washed 3 times for 10 minutes each with TBST. After washing, the membrane was probed with either a rabbit primary antiserum against pDARPP32-Thr 75 (1:500), pCaMKII (1:1000), pERK1/2 (1:2000), or pPKC (1:10000) or a mouse primary antiserum against pJNK (1:1000) for 18 hours at 4°C on a shaker. The membrane was washed again then incubated with an appropriate rabbit- (KPL, Gaithersburg, MD) or mouse secondary antiserum (BD Biosciences, CA) under the same conditions as for the primary antiserum for 1 hour at room temperature. Next, immunoreactive protein bands were detected on X-ray films using enhanced chemi-luminescence reagents (Ab Frontier, Seoul, Korea; ratio of reagents A to B = 1:500). The samples were probed for unphosphorylated proteins after stripping the same membrane that was confirmed to contain phosphorylated protein. The same membrane was also probed for β-tubulin (1:2000) to normalize the blots. Both rabbit primary and secondary antibodies against unphosphorylated proteins were diluted to 1:1000. Immunoreactive protein bands visualized on films were semiquantified using an imaging digital camera and the NIH Image 1.62 software as previously described (Choe et al., 2004). All primary antibodies were purchased from Cell Signaling Technology (Danvers, MA), except for antibodies against β-tubulin and unphosphorylated PKC (Santa Cruz Biotechnology, Inc., CA).

### Statistics

Differences in the number of immunoreactive pixels per measured area between groups following Western immunoblotting were determined by 1-way ANOVA followed by Tukey’s honestly significant difference test using GraphPad Prism 4 (GraphPad Software Incorporation, San Diego, CA). Data were expressed as the mean ± SEM for each group (n = 3–4 per group). *P*<.05 was considered to be statistically significant.

## Results

### Repeated Cocaine Administration Increases pDARPP32-Thr75 Immunoreactivity in the Dorsal Striatum

**Figure 2.**
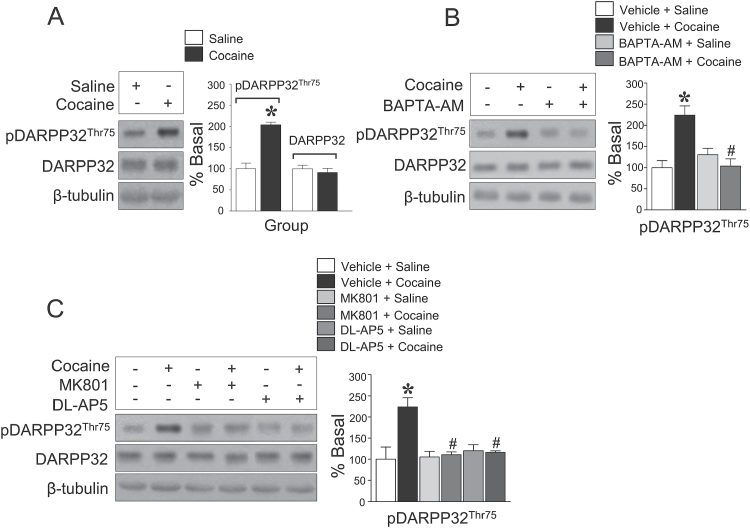
Repeated cocaine injections significantly increased pDARPP32-Thr75 immunoreactivity at 20 minutes after the final injection of cocaine as compared with unphosphorylated DARPP32 immunoreactivity (*F* = 29.05, *df* = 11) (A). Intracaudate infusion of the Ca^2+^ chelator, BAPTA-AM (*F* = 10.48, *df* = 11) (B), or the noncompetitive- and competitive NMDA receptor antagonists, MK801 (*F* = 11.39, *df* = 11) and DL-AP5 (*F* = 12.42, *df* = 11) (C), respectively, significantly decreased pDARPP32-Thr75 immunoreactivity after repeated cocaine administration. *P < .05 vs repeated saline group; *#P* < .05 vs repeated cocaine group.

We first determined whether repeated cocaine administration alters the phosphorylation of DARPP32-Thr75 in the rat dorsal striatum. To accomplish this, we surveyed changes in pDARPP32-Thr75 immunoreactivity at 20 minutes after the final saline or cocaine injection, because the activity of protein kinases is known to peak in the rat dorsal striatum at this time point (Ahn and Choe, 2010; Go et al., 2010). The results demonstrated that repeated cocaine injections significantly increased pDARPP32-Thr75 immunoreactivity compared with the control group (Figure 2A). Repeated saline and cocaine administration did not alter the immunoreactivity of unphosphorylated DARPP32-Thr75 throughout the experiments (Figure 2A). There was no change in the immunoreactivity of pDARPP32-Thr34 after repeated cocaine administration (data not shown).

### Blockade of Extracellular Ca^2+^ Influx Decreases the Repeated Cocaine-Induced Increase in pDARPP32-Thr75 Immunoreactivity

Since repeated cocaine exposure increases Ca^2+^ influx via stimulation of glutamate receptors (Ryu and Choe, 2014; Yang and Choe, 2014), we determined the contribution of Ca^2+^ influx to the phosphorylation of DARPP32-Thr75. Intracaudate infusion of the Ca^2+^ chelator, BAPTA-AM (50 nmol), significantly decreased the elevation of pDARPP32-Thr75 immunoreactivity after repeated cocaine administration (Figure 2B). Since Ca^2+^ influx is required for pDARPP32-Thr75, the involvement of NMDA receptors was also determined. Intracaudate infusion of the noncompetitive- and competitive NMDA receptor antagonists, MK801 (2 nmol) and DL-AP5 (2 nmol), respectively, significantly decreased the elevation of pDARPP32-Thr75 immunoreactivity to the basal level after repeated cocaine administration (Figure 2C).

### Blockade of Na^+^ Channels Decreases the Repeated Cocaine-Induced Increase in pDAPRR32-Thr75 Immunoreactivity but Not L-, N-, and P-Type Ca^2+^ Channels

**Figure 3.**
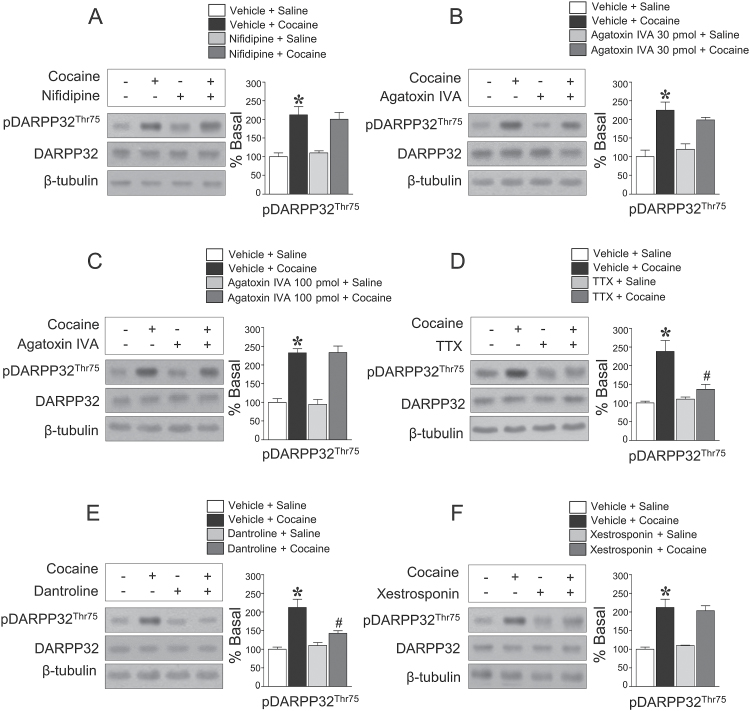
Intracaudate infusion of the L-type voltage-operated Ca^2+^-channel blocker, nifidipine (*F* = 10.61, *df* = 11) (A), the P- (*F* = 13.97, *df* = 11) (B), and N-type (*F* = 25.76, *df* = 11) (C) Ca^2+^ channel blocker, Agatoxin IVA, did not alter the repeated cocaine-induced increase in pDARPP32-Thr75 immunoreactivity. In contrast, intracaudate infusion of the Na^+^ channel blocker, TTX (*F* = 14.73, *df* = 11) (D), significantly decreased the elevation of pDARPP32-Thr75 immunoreactivity. Intracaudate infusion of the ryanodine Ca^2+^ channel blocker, dantrolene (*F* = 16.03, *df* = 11) (E), significantly decreased the repeated cocaine-induced increase in pDARPP32-Thr75 immunoreactivity, but the IP_3_ sensitive Ca^2+^ channel blocker, xestosponin (*F*= 15.53, *df*= 11) (F), did not. *P < .05 vs repeated saline group; *#P* < .05 vs repeated cocaine group.

Since NMDA receptor stimulation after repeated cocaine administration is required for DARPP32-Thr75 phosphorylation, we also determined the involvement of other Ca^2+^ channels. Intracaudate infusion of the L-type Ca^2+^ channel blocker, nifidipine (60 nmol), did not alter the repeated cocaine-induced increase in pDARPP32-Thr75 immunoreactivity (Figure 3A). Similarly, neither the P- (30 pmol) nor N-type (100 pmol) Ca^2+^ channel blocker, Agatoxin IVA, altered the level of pDARPP32-Thr75 immunoreactivity (Figure 3B-C). Since neural activity can alter pDARPP32-Thr75 via stimulation of voltage-dependent Ca^2+^ channels, the involvement of Na^+^ channels in striatal neurons was also determined. Intracaudate infusion of the Na^+^ channel blocker, TTX (1 pmol), significantly decreased the elevation of pDARPP32-Thr75 immunoreactivity after repeated cocaine administration (Figure 3D).

### Blockade of Ryanodine Ca^2+^ Channels, but Not IP_3_ Receptors, in the Endoplasmic Reticulum (ER) Decreases the Repeated Cocaine-Induced Increase in pDAPRR32-Thr75 Immunoreactivity

Stimulation of mGluRs potentiates diacylglycerol (DAG) and IP_3_ pathways via hydrolysis of phosphoinocitite (PI) in GABAergic neurons (Calabresi et al., 2014), leading to Ca^2+^ mobilization by stimulating Ca^2+^ channels in the ER. Thus, we determined the involvement of Ca^2+^ channels in the ER in this study. Intracaudate infusion of the ryanodine Ca^2+^ channel blocker, dantrolene (20 nmol), significantly decreased the repeated cocaine-induced increase in pDARPP32-Thr75 immunoreactivity (Figure 3E); however, the IP_3_-sensitive Ca^2+^ channel blocker, xestosponin C (0.004 nmol), did not (Figure 3F).

### Interactions of Protein Kinases Regulate pDARPP32-Thr75 Immunoreactivity

**Figure 4.**
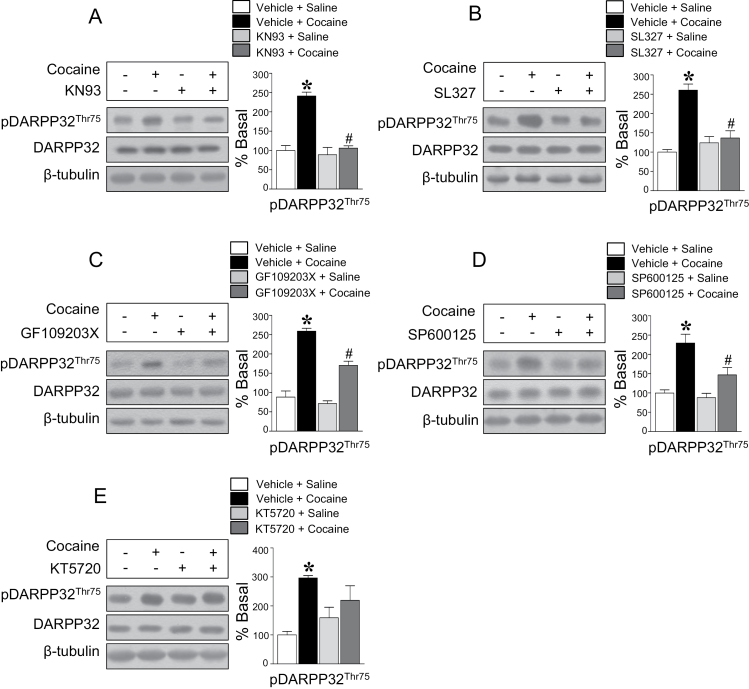
Intracaudate infusion of the CaMKII inhibitor, KN93 (*F* = 31.22, *df* = 11) (A), the ERK1/2 inhibitor SL327 (*F* = 17.43, *df* = 11) (B), the PKC inhibitor, GF109203X (*F* = 61.46, *df* = 11) (C), and the JNK inhibitor, SP600125 (*F* = 15.3, *df* = 11) (D), significantly decreased elevated pDARPP32-Thr75 immunoreactivity by repeated cocaine administration, but the PKA inhibitor, KT5720 (*F* = 4.951, *df* = 11) (E), did not. *P < .05 vs repeated saline group; *#P* < .05 vs repeated cocaine group.

Activation of Ca^2+^-dependent protein kinases and their interactions responsible for the phosphorylation of DARPP32-Thr75 were determined in this study. Intracaudate infusion of the CaMKII inhibitor, KN93 (10 nmol), significantly decreased the repeated cocaine-induced increase in pDARPP32-Thr75 immunoreactivity (Figure 4A). Similarly, the ERK1/2 inhibitor, SL327 (150 nmol), the PKC inhibitor, GF600125X (20 nmol), and the JNK inhibitor, SP600125 (200 nmol), also decreased the elevation of pDARPP32-Thr75 immunoreactivity after repeated cocaine administration (Figure 4B-D). In contrast, intracaudate infusion of the PKA inhibitor, KT5720 (5 nmol), did not alter the elevated pDARPP32-Thr75 immunoreactivity (Figure 4E).

**Figure 5.**
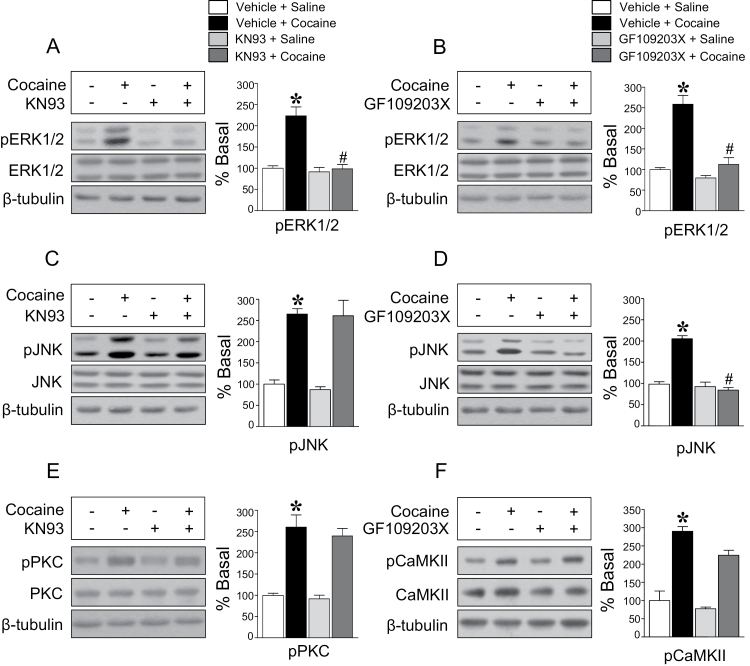
Intracaudate infusion of the CaMKII inhibitor, KN93, significantly decreased the repeated cocaine-induced increase in pERK1/2 (*F* = 16.35, *df* = 13) (A), but not pJNK (*F* = 13.76, *df* = 11) (C) and pPKC (*F* = 19.25, *df* = 13) (E) immunoreactivity. Intracaudate infusion of the PKC inhibitor, GF600125X, abolished the repeated cocaine-induced elevation of pERK1/2 (*F* = 12.65, *df* = 13) (B) and pJNK (*F* = 28.5, *df* = 13) (D), but not pCaMKII (*F* = 33.81, *df* = 13) (F) immunoreactivity. *P < .05 vs repeated saline group; *#P* < .05 vs repeated cocaine group.

Since various protein kinases phosphorylate DARPP32-Thr75, their interactions leading to the phosphorylation were also determined. Inhibition of CaMKII significantly decreased the repeated cocaine-induced increase in the immunoreactivity of pERK1/2 (Figure 5A), but not pJNK and pPKC (Figure 5C, E). Inhibition of PKC decreased the immunoreactivity of pERK1/2 and pJNK (Figure 5B, D), but not pCaMKII (Figure 5F). However, inhibition of ERK1/2 and JNK did not alter the immunoreactivity of all protein kinases surveyed in this study (data not shown).

### PPs Regulate pDARPP32-Thr75 Immunoreactivity

**Figure 6.**
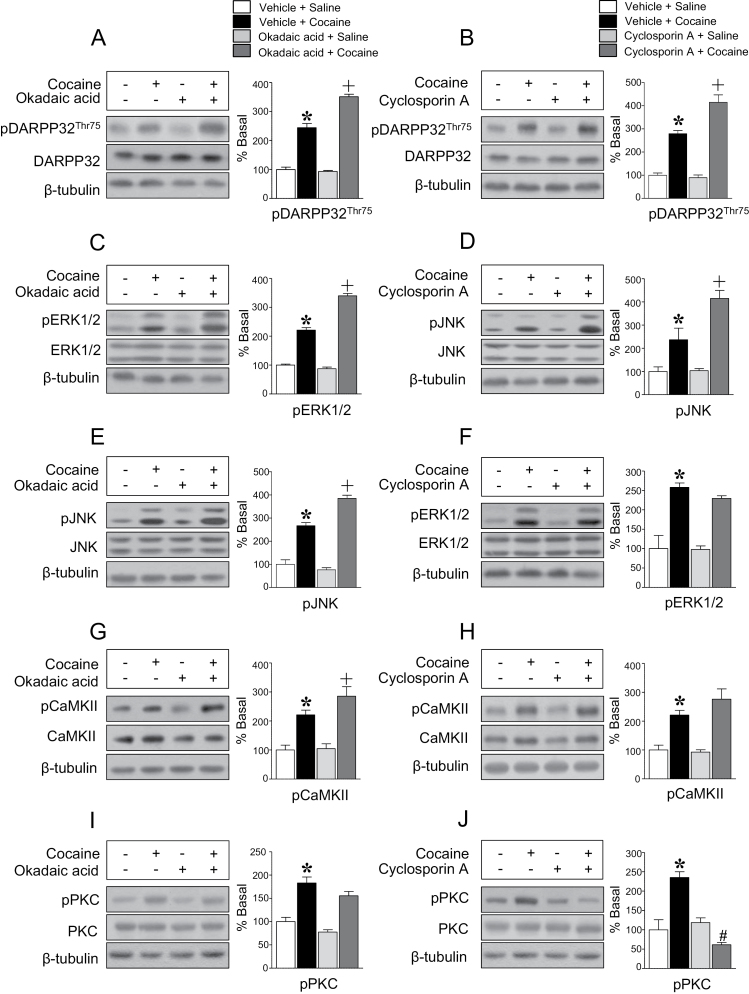
Intracaudate infusion of the PP1/2A inhibitor, okadaic acid (*F* = 136.8, *df* = 13) (A), or the PP2B inhibitor, cyclosporin A (*F* = 53.23, *df* = 13) (B), significantly increased the repeated cocaine-induced increase in pDARPP32-Thr75 immunoreactivity. In addition, intracaudate infusion of okadaic acid increased pERK1/2 (*F* = 107.0, *df* = 13) (C), pJNK (*F* = 104.3, *df* = 13) (E), and pCaMKII (*F* = 14.73, *df* = 13) (G), but not pPKC (*F* = 22.0, *df* = 13) (I) immunoreactivity after repeated cocaine administration. Intracaudate infusion of cyclosporin A also increased the repeated cocaine-induced increase in pJNK (*F* = 21.06, *df* = 11) (D), but not pERK1/2 (*F* = 24.33, *df* = 13) (F) and pCaMKII (*F* = 14.01, *df* = 13) (H) immunoreactivity. In contrasts, intracaudate infusion of cyclosporin A abolished the repeated cocaine-induced increase in pPKC immunoreactivity (*F* = 27.67, *df* = 13) (J). *P < .05 vs repeated saline group; +*P* < .05 vs repeated cocaine group; *#P* < .05 vs repeated cocaine group.

The involvement of PPs in the regulation of pDARPP32-Thr75 was determined in this study. Intracaudate infusion of the PP2/1A inhibitor, okadaic acid (0.5 nmol), significantly increased the repeated cocaine-induced increase in pDARPP32-Thr75 immunoreactivity (Figure 6A). Similar results were obtained by the intracaudate infusion of the PP2B inhibitor, cyclosporin A (0.5 nmol) (Figure 6B). Since pDARPP32-Thr75 can be regulated by both protein kinases and phosphatases, we further determined their interactions in this study. Inhibition of PP1/2A significantly increased the repeated cocaine-induced increase in the immunoreactivity of pERK1/2 (Figure 6C), pJNK (Figure 6E), and pCaMKII (Figure 6G), but not pPKC (Figure 6I). Inhibition of PP2B significantly increased the repeated cocaine-induced increase in the immunoreactivity of pJNK (Figure 6D), but not pERK1/2 (Figure 6F) and pCaMKII (Figure 6H). In contrast, PP2B inhibition abolished the repeated cocaine-induced increase in pPKC immunoreactivity (Figure 6J). Throughout a series of experiments, pretreatment with vehicle, antagonist, or inhibitor alone did not alter the immunoreactivity of phosphorylated DARPP32-Thr75 and protein kinases.

## Discussion

DARPP32 is thought to be a key molecule in drug-induced synaptic plasticity that functions by integrating dopaminergic and glutamatergic transmission in the basal forebrain (Fernandez et al., 2006). In the present study, we found that repeated cocaine administration upregulated the phosphorylation of DARPP32-Thr75 in the rat dorsal striatum. Increases in cytosolic Ca^2+^ concentrations followed by protein kinase activation through both NMDA receptors and ryanodine Ca^2+^ channels were found to be required for the phosphorylation. Moreover, activation of PPs further regulated the phosphorylation state of DARPP32-Thr75 by dephosphorylating it and downstream protein kinases. These findings suggest that glutamate receptor-induced activation of protein kinases and PPs is crucial for the regulation of pDARPP32-Thr75 after repeated cocaine exposure.

Repeated cocaine administration significantly upregulated pDARPP32-Thr75 expression in the rat dorsal striatum in this study. Previous studies have shown that repeated exposure to cocaine increases extracellular glutamate concentrations and pDARPP32-Thr75 in the rat dorsal striatum (Scheggi et al., 2004). Moreover, stimulation of group I mGluRs (mGluR1/5) with DHPG is known to increase pDARPP32-Thr75 in neostriatal slices of C57/BL6 mice (Liu et al., 2001). These findings suggest that elevation of glutamate and subsequent glutamate receptor stimulation are necessary for the phosphorylation of DARPP32-Thr75 after repeated cocaine administration. Increases in pDARPP32-Thr75 after repeated cocaine administration were significantly decreased by preventing Ca^2+^ influx in the present study, suggesting that activation of Ca^2+^ channels is required for the phosphorylation. Blockade of NMDA receptors, but not L-, N-, and P-type Ca^2+^ channels, abolishes the repeated cocaine-induced increase in pDARPP32-Thr75, indicating that NMDA receptors may act as primary Ca^2+^ channels for the phosphorylation. However, the possibility that other Ca^2+^ channels can contribute to the phosphorylation at other time points should not be excluded because L-type Ca^2+^ channels have the capability to produce Ca^2+^ cascades in a very short-time period in GABAergic neurons (Brittain et al., 2012).

Blockade of Na^+^ channels attenuates the elevation of pDARPP32-Thr75 after repeated cocaine exposure, suggesting that neural activity caused by stimulating Na^+^ channels contributes to DARPP32-Thr75 phosphorylation. It is well known that stimulation of Na^+^ channels in glutamatergic terminals in response to cocaine results in stimulation of voltage-operated Ca^2+^ channels, leading to increase in glutamate release in the striatum (Swanson et al., 2001). This increase stimulates Na^+^ channels such as AMPA and kainate glutamate receptors in GABAergic striatal neurons and produces excitatory postsynaptic potentials, resulting in the elevated release of Ca^2+^ from the internal stores (Di Ciano and Everitt, 2001; Vanderschuren et al., 2005; Robison et al., 2013). Taken together, increases in membrane potentials by increasing Ca^2+^ and Na^+^ influxes in GABAergic neurons after repeated cocaine further increase cytosolic Ca^2+^ concentrations via voltage-operated Ca^2+^ channels in the ER (Ryu and Choe, 2014), leading to DARPP32-Thr75 phosphorylation. Thus, blockade of ryanodine-sensitive Ca^2+^ channels in the ER decreased the elevation of the phosphorylation in this study. A previous study have shown that stimulation of mGluR1/5 upregulates pDARPP32-Thr75 via signaling cascades linked to phospholipase C (PLC)/CDK5 in mice (Yamamura et al., 2013). These findings suggest that activation of the PLC pathway leading to CDK5 rather than IP_3_ is more effective to evoke the phosphorylation. In addition to CDK5, PKC can be activated by activation of both NMDA receptors and mGluRs via increases in Ca^2+^ influx and PI hydrolysis, respectively (Liao et al., 2001; Liu et al., 2001; Oh et al., 2013; Yamamura et al., 2013). Thus, activation of PKC linked to glutamate receptors drives Ca^2+^ mobilization, which is likely necessary for the phosphorylation of DARPP32-Thr75 in response to repeated cocaine exposure.

Increases in cytosolic Ca^2+^ concentrations activate Ca^2+^-dependent protein kinases (Pierce et al., 1998; Beninger and Gerdjikov, 2004), which suggests that a glutamate receptor-stimulated increase in Ca^2+^ followed by activation of protein kinases is responsible for the phosphorylation of DARPP32-Thr75 after repeated cocaine administration. Interactions of protein kinases in turn regulate the phosphorylation state of DARPP32-Thr75 in a complicated manner. In the present study, inhibition of CaMKII following repeated cocaine administration was found to reduce ERK1/2, but not PKC and JNK phosphorylation. Inhibition of PKC reduces ERK1/2 and JNK, but not CaMKII, phosphorylation. However, inhibition of either JNK or ERK1/2 does not influence any other protein kinases. These findings suggest that increases in cytosolic Ca^2+^ mobilization after glutamate receptor stimulation are crucial for phosphorylation of DARPP32-Thr75 through the activation of PKC/JNK, PKC/ERK1/2, and CaMKII/ERK1/2. As an upstream transducer of pDARPP32-Thr75, ERK1/2 can be phosphorylated by the stimulation of dopamine D1 receptors after repeated cocaine administration (Valjent et al., 2000). Stimulation of dopamine D1 receptors is found to potentiate the cAMP/PKA cascades, which activates ERK1/2 (Grewal et al., 1999; Murer and Moratalla, 2011). Activation of cAMP/PKA cascades also activates ERK1/2 through phosphorylation of NMDA receptors in which catalytic activity of cAMP may be PKA-dependent or PKA-independent in the dorsal striatum (Roberson et al., 1999; Choe and Wang, 2002; Cahill et al., 2014).

Activation of PPs controls the phosphorylation state of signaling molecules after cocaine administration (Kim et al., 2009). In this study, inhibition of PPs themselves did not alter the phosphorylation state of DARPP32-Thr75 as well as protein kinases, while it synergistically upregulated the phosphorylation after cocaine exposure. These findings suggest that the basal activity of PPs is low, but once activated, they have capacity to dephosphorylate protein kinases with different sensitivity. For instance, the counter activity of PP1/2A against protein kinases is greater than that of PP2B, as demonstrated by the finding that inhibition of PP1/2A significantly increases protein kinase activity to a greater degree than PP2B. These findings suggest that the phosphorylation state of DARPP32-Thr75 is more affected by PP1/2A activation after repeated cocaine. In addition, activation of both PP1/2A and PP2B more effectively deactivated pJNK, suggesting that JNK plays an important role in control of the phosphorylation state of pDARPP32-Thr75. However, the spatiotemporal kinetics between protein kinases and phosphatases have yet to be identified. Unlike dephosphorylation of other protein kinases, PP2B has been found to activate PKC, suggesting the presence of upstream regulators linked to PKC. Even though supporting evidence is limited, it is clear that activation of PPs after repeated cocaine administration further regulates the phosphorylation state of DARPP32-Thr75 in the dorsal striatum.

## Conclusion

**Figure 7.**
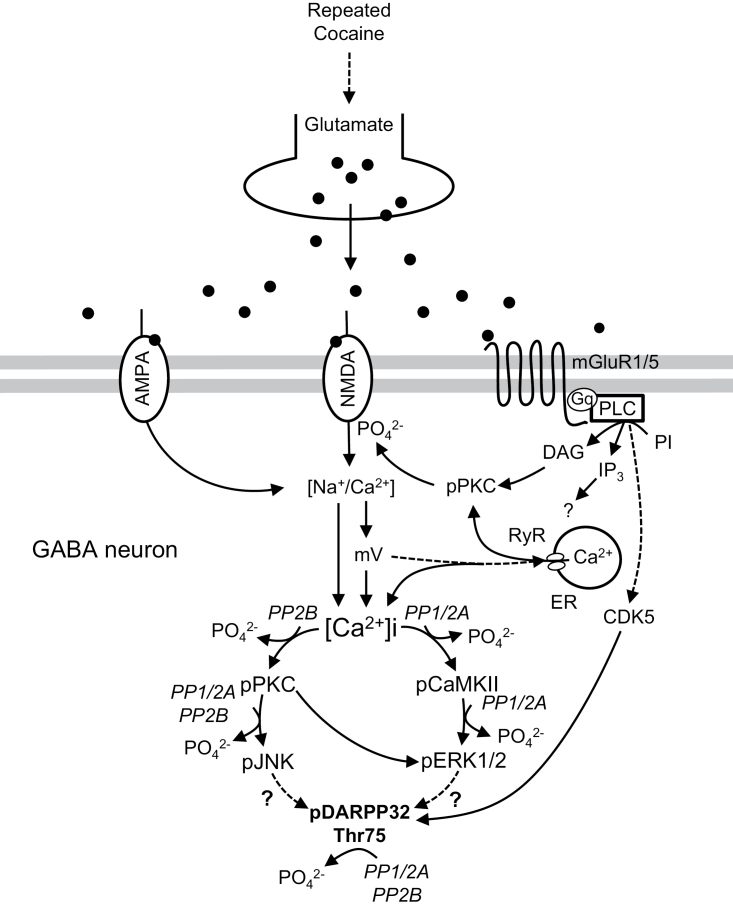
A postulated diagram illustrating pharmacological mechanisms underlying repeated cocaine-stimulated phosphorylation of DARPP32-Thr75 via stimulation of the glutamate receptor-mediated Ca^2+^ increase followed by activation of protein kinases and PPs. PPs further regulate the phosphorylation state of DARPP32-Thr75 in a protein kinase-specific manner. Putative interactions are discussed in detail in the text. Solid arrows represent the facilitatory regulation of downstream targets, while broken arrows represent indirect stimulation of the targets. [Ca^2+^], Ca^2+^ concentration; p, phosphorylation; mV, membrane potential; RyR, ryanodine receptor.

Repeated cocaine increases Ca^2+^ mobilization and leads to the phosphorylation of DARPP32-Thr75 in the rat dorsal striatum. As postulated in Figure 7, repeated cocaine increases extracellular glutamate concentrations through the basal ganglia circuitry (Kalivas, 2004). This increase stimulates both ionotropic glutamate receptors and mGluRs, and their interactions upregulate extracellular Ca^2+^ as well as Na^+^ influxes into the GABAergic neurons (Kalivas, 2004). Increases in the ion influxes subsequently increase excitatory postsynaptic potentials, leading to the release of Ca^2+^ from the ER. This increase potentiates the activation of Ca^2+^-dependent protein kinases including PKC, CaMKII, ERK1/2, JNK, and upstream regulators of PKC. Protein kinase interactions such as PKC/JNK, PKC/ERK1/2, and CaMKII/ERK1/2 may result in DARPP32-Thr75 phosphorylation, probably via downstream intermediates affected by JNK and ERK1/2. In addition, PPs activated by the stimulation of glutamate receptors further regulate the phosphorylation state by dephosphorylating pDARPP32-Thr75 and protein kinases. Taken together, dynamic control of the phosphorylation state of DARPP32-Thr75 by protein kinases and phosphatases seems to be a key physiological event in cocaine-induced synaptic plasticity in the dorsal striatum.

## Statement of Interest

None.
